# Predicting Structure and Transport in Disordered Mesoporous Materials via Cooperative Phase Transitions

**DOI:** 10.1021/acsmaterialsau.5c00213

**Published:** 2025-12-25

**Authors:** Georgiy Baroncha, Eustathios S. Kikkinides, Theresa Paul, David Poppitz, Dirk Enke, Rustem Valiullin

**Affiliations:** † Felix Bloch Institute for Solid State Physics, 9180 Leipzig University, Linnéstr. 5, 04103 Leipzig, Germany; ‡ Department of Chemical Engineering, 37782 Aristotle University of Thessaloniki, 54124 Thessaloniki, Greece; § Institute for Chemical Technology, Leipzig University, Linnéstr. 3, 04103 Leipzig, Germany

**Keywords:** gas sorption, cooperative phase transitions, pore networks, diffusive transport, pore structure

## Abstract

The interplay between material structure, thermodynamics, and transport of confined fluids in nanoporous solids underpins their practical applications. Mesoporous networks embedded within microporous frameworks of zeolites and MOF materials are attracting increasing attention as they can enhance material properties and boost their performance. Correlating the mesoporous network structure with transport properties, however, remains challenging due to an apparent conflict: most thermodynamic models focus on single-pore equilibrium behavior, whereas transport is largely dictated by the organization of the pore network. Herein, we show that exploiting cooperative phenomena in gas adsorption governed by structural disorder resolves this challenge. We present a unified framework that links the structure, thermodynamics, and transport by leveraging recent advances in the statistical thermodynamic description of nonequilibrium-phase states arising from cooperativity across pore networks. Structural descriptors of the mesopore space, extractable from gas sorption measurements including, beyond conventional pore size distributions, the average pore connectivity and a hierarchy factor describing deviations from a fully random structure, are used to accurately predict diffusive transport. The framework’s robustness is validated for mesoporous materials with both homogeneous and hierarchical pore architectures through experiments using transmission electron microscopy (TEM), mercury intrusion, and pulsed-field gradient (PFG) NMR.

## Graphical abstract

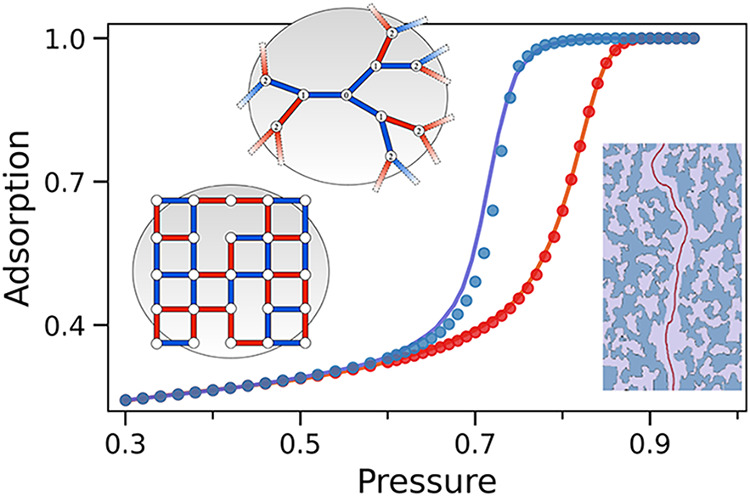

## Introduction

Porous solids are widely used in applications such as catalysis, drug delivery, oil and gas recovery, and mass separation.^1^ The functional properties of these materials, particularly molecular transport, and chemical composition are strongly influenced by the geometry of their pore space. Accurate textural characterization is therefore essential for understanding the behavior of confined materials, quantitatively predicting the performance and guiding chemical synthesis to tailor structural properties. Among the available experimental techniques, gas sorption occupies a special place due to its straightforward implementation, making it a routine method in both research and industry. Because of its widespread use, significant progress has been made in understanding gas–liquid equilibria in pore systems with simple geometries. However, the role of pore interconnectivity in gas sorption remains largely neglected, limiting current approaches to oversimplified structural models, thus impeding both the rational tailoring of the structure and its correlation with functional performance.^2,3^ This gap is particularly critical because most industrially relevant porous materials have a highly complex pore structure.^4^

Molecular transport is a fundamental property that strongly influences the performance of a wide range of processes. Optimizing diffusive transport in porous materials through various strategies, especially hierarchical structuring, is a major focus of current research across many fields.^5–11^ Introducing mesopore networks into zeolites,^12–15^ carbons,^16,17^ and nowadays into MOFs^15,18–23^ has been shown to greatly improve their transport properties and increase their functionalities. Establishing rigorous relationships between transport properties and structural descriptors accessible from adsorption–desorption measurements would provide significant advantages. However, most current models used in gas sorption are restricted to equilibrium thermodynamic descriptions of phase behavior in individual pores, which overlooks the inherently multipore nature of diffusion processes. Numerous studies have shown that diffusion is also governed by properties such as pore connectivity, disorder correlation length, and pore hierarchy.^24,25^ Advancing our understanding of phase equilibria in complex pore systems is therefore essential for elucidating their transport behavior and, more generally, providing a rational basis for correlating the structure with various physical properties.

The independent domain or independent pore model (IPM) remains a cornerstone approach for analyzing gas sorption data by reducing the complexity of the pore space to simple collections of idealized pores.^26^ The phase equilibria in individual pores can be determined very accurately using the concepts of equilibrium thermodynamics.^27^ The adsorption/desorption transitions are obtained as the cumulative sum of the isotherms of individual pores, described by the kernels *K*(*x*, *p*)­

$$V_{a}\left(p\right)=\int K\left(x,p\right)\phi \left(x\right)dx$$ 1

where *V*_a_ is the volume adsorbed at pressure *p*, *x* is the pore size, and ϕ­(*x*) is the volumetric pore size distribution (PSD).

It is well established that the system states encountered along isotherms in pore networks can strongly deviate from equilibrium due to interpore connectivities, which give rise to cooperative effects spanning multiple pores.^28–32^ The latter are caused by a subtle interplay between two key processes: (i) the nucleation of new phases, i.e., liquid bridging during adsorption and gas bubble cavitation during desorption and (ii) the subsequent percolative growth of these newly formed domains and/or the phase supplied at the pore boundaries. The intermixing of nucleation and percolative growth processes, as dictated by pore network architecture, disrupts the one-to-one correspondence between the externally controlled gas pressure and phase state within individual pores.^28^ This has far-reaching consequences with the most critical one being that the commonly used eq 1 becomes invalid.

Neglecting cooperativity effects in the structure analysis may lead to significant structural misinterpretations. Therefore, numerous studies have attempted to capture network effects, most prominently through percolation theories applied to desorption.^33–42^ By contrast, connectivity effects during adsorption have been largely overlooked, with only a few notable exceptions.^43,44^ Notably, however, no approach has yet been available that provides a common model accounting for network effects in both adsorption and desorption.

As noted previously, eq 1 fails to capture phase equilibria accurately in complex pore spaces. A more general framework, valid for both the IPM and interconnected pore networks, has recently been introduced.^45^ It is based on partitioning PSD ϕ­(*x*) into two distribution functions representing the portions of ϕ­(*x*) filled with capillary-condensed, ψ_f_(*x*, *p*), and gaseous, ψ_g_(*x*, *p*), phases

$$V_{a}\left(p\right)=\int V_{p}\left(x\right)\psi_{f}\left(x,p\right)dx+\int V_{af}\left(x,p\right)\psi_{g}\left(x,p\right)dx$$ 2

In eq 2, *V*_p_(*x*) and *V*_af_(*x*, *p*) are the pore and adsorbed film volumes, respectively.

In this formulation, all structural network details are reflected in ψ_f_(*x*, *p*) and ψ_g_(*x*, *p*). The statistical thermodynamic approach for deriving these distribution functions for any state within the hysteresis region was first demonstrated for the statistically disordered chain model (SDCM).^45^ The latter is a primitive form of a disordered pore network model with serially connected pores with statistically distributed pore sizes, yielding pore connectivity of *z* = 2. It provided the first self-consistent study in which the interplay of nucleation and invasive growth processes was linked to the network structure through statistical thermodynamics. This framework was later extended to infinite Bethe lattices (BL) with connectivities *z* >2 with statistically distributed pore sizes (SDBL) (ref 46). In this model, lattice bonds were modeled as long cylindrical pores, and a fraction *f*_0_ of randomly selected bonds was designated as surface pores exposed to the gas phase.^40^ The solution involved transcendental equations containing polynomials of integer degree *z*, where *z* is the lattice connectivity.^35,40,46^ These developments, which advance gas sorption analysis toward probing network properties,^47,48^ can substantially contribute to improving transport modeling in porous materials. However, to establish a link between gas sorption and transport, several critical questions must first be addressed. These include, in particular, the physical meaning of fractional connectivities obtained from inverse problem solutions,^47^ which features of network architecture that govern diffusive transport are captured in sorption isotherms, and whether loopless Bethe structures can reliably reproduce the sorption behavior of regular networks with reconnections.

These questions have recently been addressed by considering Bethe lattices with statistically distributed connectivities.^49^ Such networks, known as Galton–Watson trees,^50^ extend Bethe lattices by introducing random branching at each lattice node, as illustrated in Figure 1. Phase equilibria in these statistically disordered Galton–Watson (SDGW) lattices were analyzed using the framework developed earlier,^46^ which revealed several key findings: (i) the analytical framework proposed in ref 46 can be reliably employed to solve the inverse problem in gas sorption for SDGW structures,^47^ and the pore connectivity treated as a variable in the numerical minimization procedure represents the number-average connectivity of the entire pore network; (ii) sorption behavior is largely independent of the specific connectivity distribution for networks with the same average connectivity, as demonstrated in Figure 2A–C; (iii) the phase behavior in Bethe-like and regular networks with the same average connectivity is nearly identical, as illustrated in Figure 2D–F. These results demonstrate that SDGW lattices—and therefore the theory presented in ref 46—can reliably capture phase coexistence in real porous materials. This finding suggests that gas sorption measurements, when combined with the statistical thermodynamic theory for Bethe lattices, enable reliable extraction of the pore size distribution, average pore connectivity, and fraction of boundary pores via an inverse problem solution, thereby laying the foundation for transport modeling.

**Figure 1:**
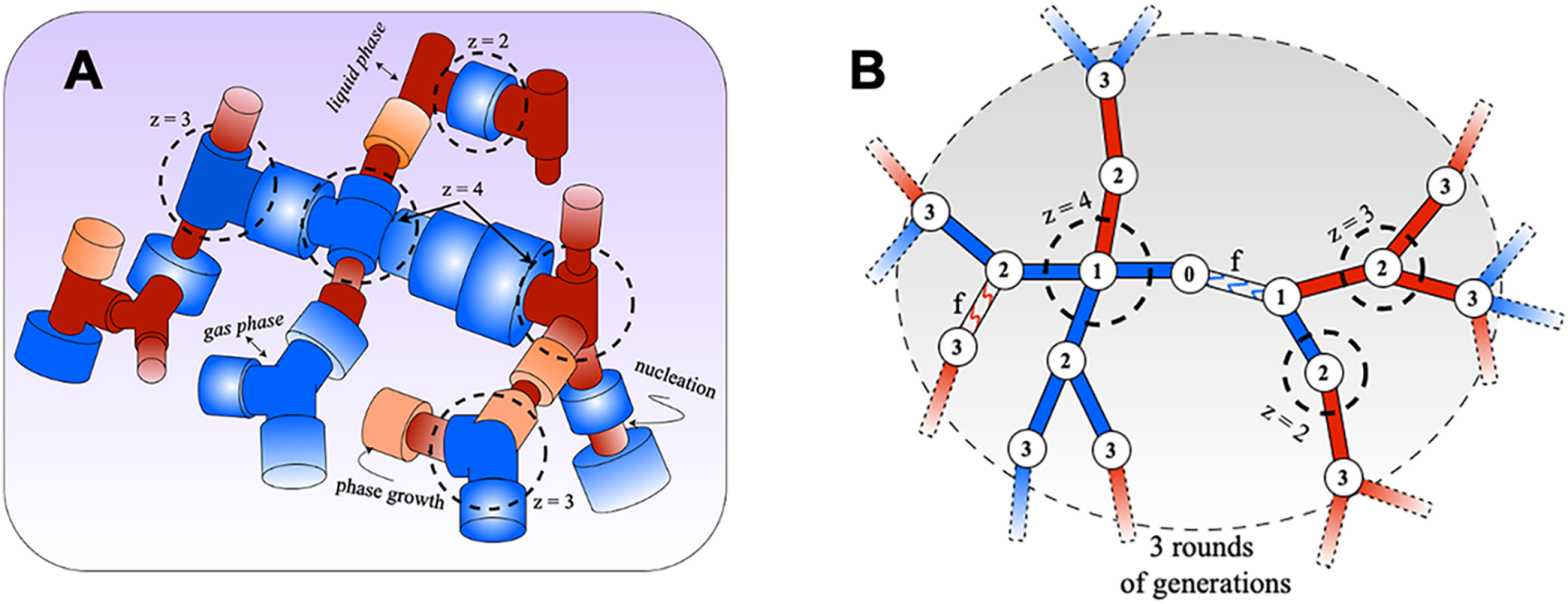
Unifying structural model to link gas sorption with transport. (A) Schematic representation of the random network structure used to model adsorption and transport properties. Each bond is modeled as a long cylindrical pore and connects at nodes to *z* – 1 neighbors, with *z* randomly drawn from a prescribed connectivity distribution function. Channel sizes are statistically distributed according to the pore size distribution function ϕ­(*x*). Colors illustrate phase coexistence exemplified for desorption: liquid (red and orange sections) and gas (blue sections). In the section indicated in orange, gas–liquid transition is initiated via capillary liquid bridging (nucleation), followed by invasion (phase growth) into adjacent sections. (B) Example of the first three generations of a computer-generated Galton–Watson tree representing the pore network structure. Node numbers indicate the generation.

**Figure 2:**
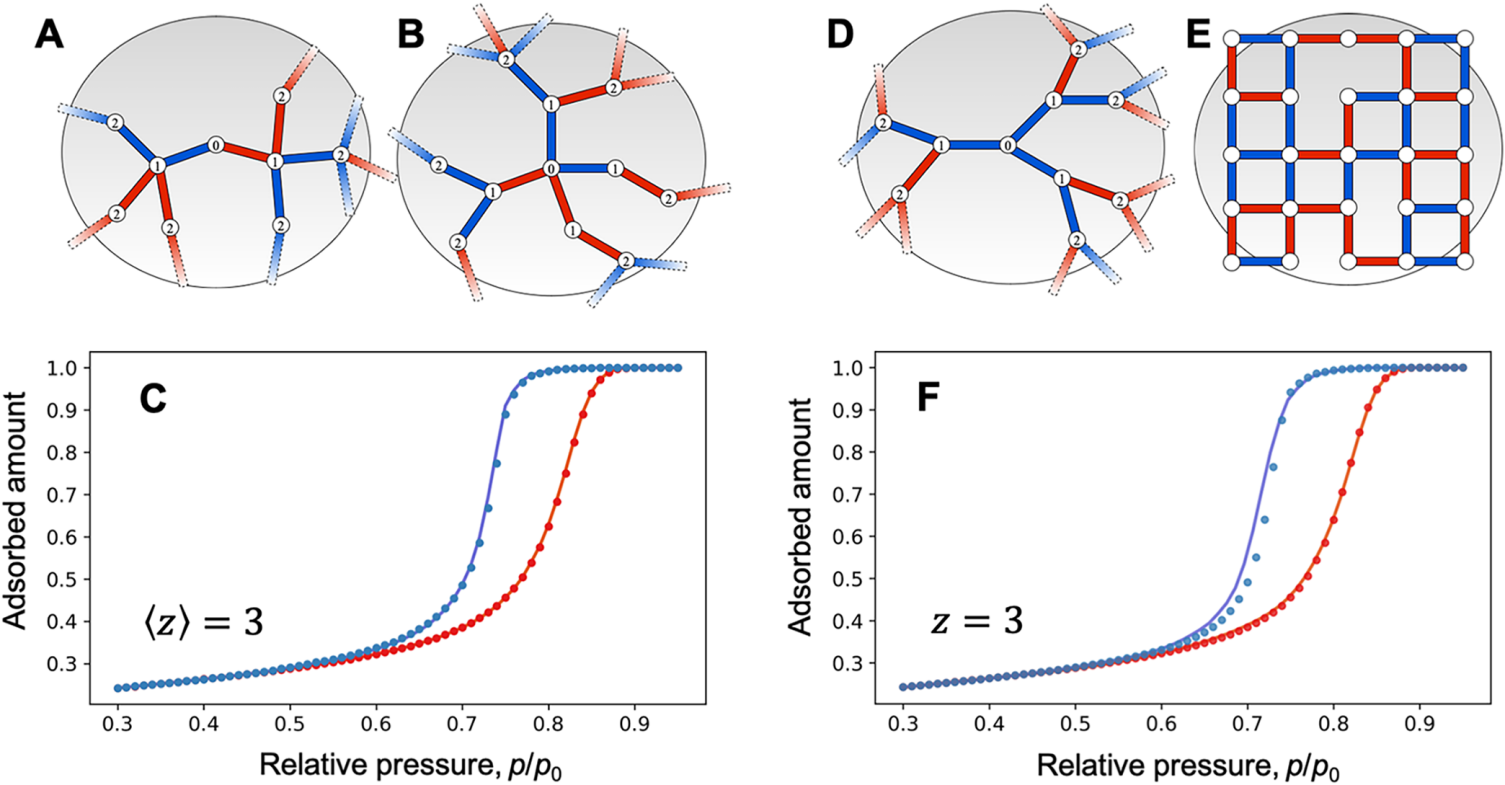
Influence of network topology and reconnections on sorption behavior. (A, B) Two networks with the same average connectivity, ⟨*z*⟩ = 3, but with different connectivity distribution functions, χ­(*z*) = [1/2; 0; 1/2] and [1/3; 1/3; 1/3], respectively. (C) Corresponding sorption isotherms, which are nearly indistinguishable. (D, E) Bethe and regular lattices with *z* = 3. (F) Their sorption isotherms are also nearly identical. Bond colors indicate phase coexistence between liquid (red bonds) and gas (blue bonds) phases.

Theoretical modeling of diffusive transport using various network models has a long and rich history.^24^ Keeping in mind the connection to gas sorption, transport on disordered branching networks, such as Bethe lattices, has been thoroughly analyzed.^51^ As discussed by Sahimi,^52^ these types of models can be useful, while their great advantage is that they are susceptible to exact analytical treatments and may be valid for porous solids with broad PSDs, especially for networks with low connectivities.^53^ The effective medium approximation (EMA) offers significant simplifications in computations, while preserving the high accuracy.^52,54^ The results obtained through EMA can be further extended to materials with two-pore level or hierarchical organizations.^55–57^

The apparent conflict between single-pore thermodynamics and multipore transport has historically led to their independent treatment. In particular, the lack of comprehensive structural descriptors has hindered the foundation of meaningful structure–transport correlations. In this work, we present a unified framework that directly links gas sorption and diffusive transport through a common structural model. Leveraging recent advances in the statistical thermodynamic description of cooperative phase transitions, we identify three key structural parameters that fully govern gas–liquid coexistence in complex pore networks, including the PSD, average pore connectivity, and fraction of boundary pores or hierarchy factor, which enable reliable modeling of diffusive transport. We demonstrate that these parameters, extracted from nitrogen gas sorption measurements on random porous glasses, can predict transport in materials with both homogeneous and hierarchical pore structures by using established theoretical transport models. These predictions are further validated by PFG NMR diffusion measurements and structural analyses based on transmission electron microscopy (TEM) and mercury intrusion. This unified framework establishes a direct, synergistic connection between thermodynamic and dynamic properties of complex porous materials, enabling the rational design and synthesis of materials with tailored diffusive performance, which until now has been governed by empirical trial and error procedures.^58^

The remainder of the paper is organized as follows. First, we recapitulate the key aspects of our previous work by presenting the statistical thermodynamic framework developed to trace phase transitions in Bethe lattices with statistical disorder.^45,46^ We then demonstrate that this analytical framework accurately captures phase transitions in complex pore structures, including Bethe lattices with randomly distributed connectivities and random regular lattices with reconnections.^49^ Thereafter, we present a methodology for solving the inverse problem to extract pore size distributions (PSDs), pore connectivities, and fractions of boundary pores from nitrogen gas sorption data and apply it to two random porous glass materials. Next, we adopt Stinchcombe’s theory of conductivity in Bethe lattices with average fractional connectivities and apply it to predict diffusive transport using structural descriptors obtained from gas sorption analysis. Using PFG NMR, we demonstrate that while this prediction is excellent for one material, it fails significantly for the other. We further show that this discrepancy arises from structural correlations, specifically hierarchical pore-space organization in the latter material, and validate this conclusion using TEM and mercury intrusion. We argue that the original interpretation of the fraction of boundary pores should be revised to account for hierarchical organization. Finally, we demonstrate that applying composite media theories for diffusive transport, incorporating a hierarchy factor extracted from gas sorption data, leads to excellent agreement between experimental observations and theoretical predictions.

## Experimental Section

### Materials

In this work, we used two borosilicate glasses: Vycor, a sodium borosilicate (72.5/20.5/7 mol % SiO_2_/B_2_O_3_/Na_2_O), and KBS-A, a potassium borosilicate (50/43/7 mol % SiO_2_/B_2_O_3_/K_2_O) with high borate content. Because potassium ions have a larger ionic radius and diffuse more slowly than sodium ions, KBS-A undergoes a slower and more controllable phase separation. This limits domain growth and coarsening, enabling better morphological control even during cooling from the melt. Its higher borate content also produces larger borate-rich domains, which, in turn, result in a higher pore volume after leaching compared to Vycor porous glass. KBS-A glass was synthesized by stoichiometrically mixing SiO_2_, B_2_O_3_, and K_2_CO_3_ in a ball mill at 450 rpm for 5 min. To compensate for boron oxide volatilization during melting, an additional 3 wt % of B_2_O_3_ was added to the batch. The nominal composition of KBS-A was 50 mol % SiO_2_, 43 mol % B_2_O_3_, and 7 mol % K_2_O. The mixture was melted at 1450 °C, then cooled, crushed, and remelted to ensure compositional homogeneity. The homogenized melt was cast onto a preheated brass plate and slowly cooled to room temperature. The resulting glass was crushed and sieved to obtain granules with particle sizes between 1 and 2.5 mm. These granules were leached in distilled water for 4 days and subsequently dried.

### Nitrogen Gas Sorption

For nitrogen sorption analysis 100 mg of sample was degassed at 250 °C for 16 h under ultrahigh vacuum. The measurements were performed on a Autosorb iQ from Quantachrome Instruments/Anton Paar at a temperature of 77 K in the relative pressure range of 0–0.995 *p*/*p*_0_. For acquiring the isotherm, adsorption points were measured at 0.025 *p*/*p*_0_ intervals and desorption points from 0.995 to 0.1 *p*/*p*_0_ with an interval of 0.0125 *p*/*p*_0_ between points. Equilibration time at high relative pressures was 6 min. The total pore volume was determined on the adsorption branch of the isotherm at a relative pressure of 0.95 *p*/*p*_0_ (Figure 3).

**Figure 3:**
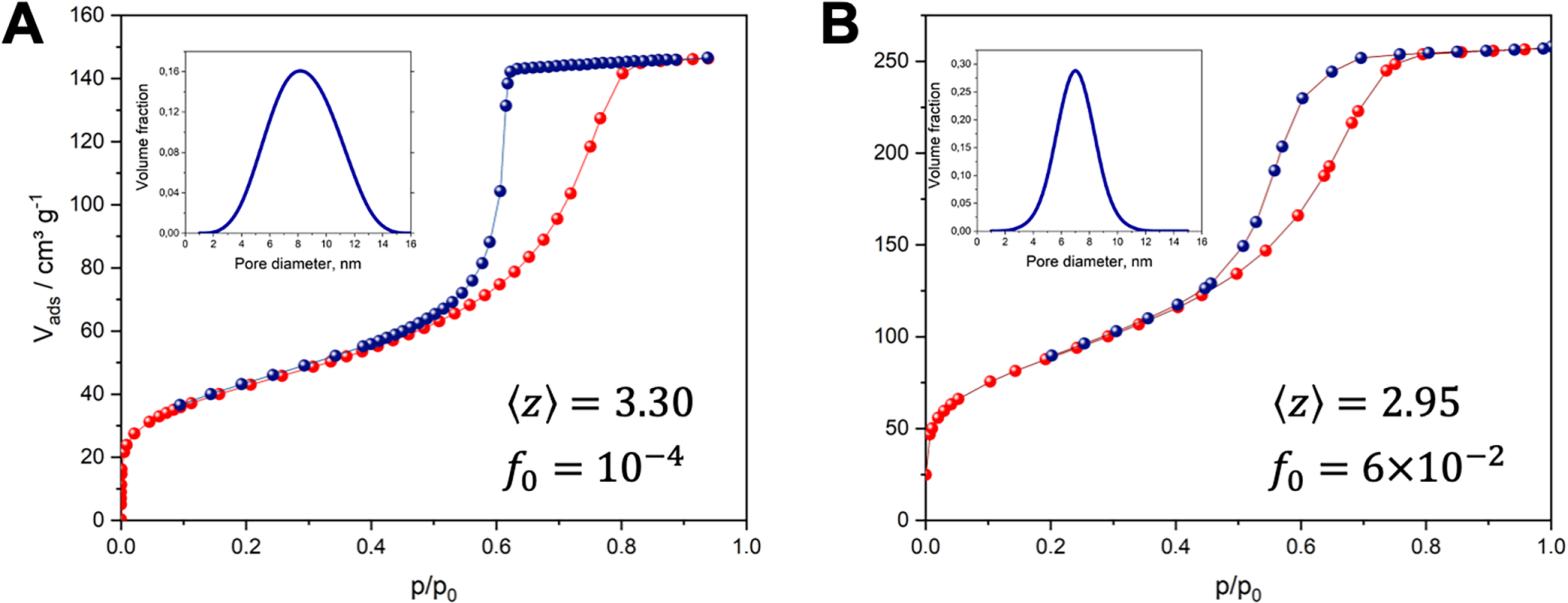
N_2_ adsorption behavior and structural characterization of mesoporous glasses. (A, B) N_2_ adsorption and desorption transitions at 77 K obtained for Vycor porous glass and KBS-A, respectively. Insets display the volumetric pore size distributions ϕ­(*x*), average connectivities ⟨*z*⟩, and surface pore fractions *f*_0_ extracted from the inverse problem solution of eq 2.

### Pulsed-Field Gradient NMR

For the measurements, the porous materials were first outgassed at a high temperature. Afterward, normal *n*-hexane was added in excess without contact with air. All experiments were performed using a Bruker spectrometer operating at 400 MHz proton resonance frequency and equipped with a Diff50 diffusion probehead. The stimulated echo pulse sequence with short diffusion times *t*_d_ = 6 ms was used. The measurement temperature was 15 °C.

### Transmission Electron Microscopy

Transmission electron microscopy (TEM) was performed using a JEOL JEM-2100Plus instrument, operated at an accelerating voltage of 200 kV. The transmission electron microscope is equipped with a LaB6 cathode and a high-resolution pole piece, enabling a point resolution of 0.23 nm in the TEM mode. Images were recorded by using a TVIPS ultrafast 4K CMOS camera. The samples were prepared by grinding them in ethanol and transferring the dispersed particles to a holey carbon Cu-TEM grid after segregation.

### Mercury Intrusion

The samples were analyzed by mercury intrusion porosimetry, where they were degassed at 0.2 mbar and RT with a Pascal 140 porosimeter. Mercury intrusion was performed with a Pascal 440 porosimeter from Thermo Scientific in a pressure range of 1–1000 bar to probe only the large pores contained in the sample. Mercury surface tension was assumed to be 0.484 N m^–1^, and the contact angle was set to 141.3°. The pore width distribution was calculated with the Washburn equation, whereby cylindrical pores were assumed.

## Theoretical Section

In this section, we summarize the most essential elements of the statistical thermodynamic framework used to model gas sorption in random pore networks, with the aim of clarifying which structural descriptors can be accessed and how they are obtained (see refs 45,46,49 for theoretical details). As mentioned earlier, the key task here is to compute the two distribution functions ψ_f_(*x*, *P*) and ψ_g_(*x*, *P*) in eq 2. For the IPM, following the spirit of eq 1, these functions are obtained straightforwardly by determining the pressure-dependent critical pore diameters for liquid bridging during adsorption and for the equilibrium gas invasion during desorption. These critical pore diameters partition the PSD into two regions, which correspond to the distribution functions of interest.

In contrast, when network effects are considered, the interplay between different transition mechanisms must be taken into account. This interplay depends on the architecture of the pore space, which we model using Bethe lattices characterized by a coordination or connectivity number *z*. In these lattices, the bonds represent long cylindrical pores of equal length, and their diameters *x* follow a volumetric PSD ϕ­(*x*) without correlations between the pore sizes of adjacent bonds. Because we consider infinite Bethe lattices, the occurrence of boundary pores having contact with the external gas phase is modeled, following Pinson et al.,^40^ by introducing a parameter *f*_0_, which denotes the fraction of pores (bonds) randomly distributed throughout the network and assigned as connected to the external bulk phase.

We consider two mechanisms for capillary condensation and evaporation. The first mechanism, *phase growth*, corresponds to phase transitions occuring at equilibrium and involving the invasion of a phase already present at the pore boundaries. The second mechanism, *nucleation*, involves the spontaneous formation of a new phase within a pore, through either cavitation (gas nucleation) or the formation of liquid bridges (liquid nucleation).

For simplicity, the junctions in the lattices are considered to be volumeless; their role is solely to impose thermodynamic boundary conditions on the connected pores. For adsorption, the junction is regarded as filled if (*z* – 1) of its pores are already filled with liquid. In this case, the remaining *z*-th pore, still containing gas, acquires a boundary contact with the liquid phase. Once the junction is filled with liquid, metastability is also removed for the last remaining pore, which can fill now by phase growth. During desorption, the junction is considered empty if at least one of its *z* pores contains gas. In the latter case, the gas phase removes the metastability for the junction, causing it to empty at the same pressure. Consequently, all remaining (*z* – 1) pores connected to this junction gain boundary contact with the gas phase and may empty upon gas invasion. For a more detailed discussion of these rules, see ref 46.

Adsorption transition is treated using two probabilities, *P*_n,f_ and *P*_g,f_, for filling the pores via nucleation

3 $$P_{n,f}=\frac{\int_{0}^{x_{n}}x^{-2}\phi \left(x\right)dx}{\int_{0}^{\infty }x^{-2}\phi \left(x\right)dx}$$

and phase growth

4 $$P_{g,f}=\frac{\int_{0}^{x_{g}}x^{-2}\phi \left(x\right)dx}{\int_{0}^{\infty }x^{-2}\phi \left(x\right)dx}$$

where *x*_n_ and *x*_g_ are the critical pore diameters for liquid bridging and equilibrium filling, respectively.

The probability *I* that a pore is filled with liquid by any of the two mechanisms during adsorption has been shown to satisfy a transcendental equation^46^

5 $$I=P_{n,f}+\left(P_{g,f}-P_{n,f}\right)\cdot (\left(1-f_{0}\right)I{)}^{z-1}$$

It is related to the probability *P*_tr,f_(*p*, *z*, *f*_0_)­

6 $$P_{\mathrm{tr},f}=1-{\left(1-I^{z-1}\right)}^{2}$$

that a randomly selected pore is connected directly to at least one filled junction and hence can be filled by phase growth.

Finally, the part of filled pores ψ_f_(*x*, *p*) for the case of statistical disorder becomes

7 $$\psi_{f}\left(x,p\right)=\{\begin{array}{ll} \phi \left(x\right), & x\leq x_{n}\\ P_{tr,f}\left(p,z,f_{0}\right)\cdot \phi \left(x\right), & x_{n}<x\leq x_{g}\\ 0, & x_{g}<x \end{array}$$

Desorption analysis is based on the probabilities, *P*_n,e_ and *P*_g,e_, for a pore to empty via cavitation or equilibrium gas invasion, respectively. They are defined as

8 $$P_{n,e}=\frac{\int_{x_{c}}^{\infty }x^{-2}\phi \left(x\right)dx}{\int_{0}^{\infty }x^{-2}\phi \left(x\right)dx}$$

and

9 $$P_{g,e}=\frac{\int_{x_{g}}^{\infty }x^{-2}\phi \left(x\right)dx}{\int_{0}^{\infty }x^{-2}\phi \left(x\right)dx}$$

where *x*_c_ and *x*_n_ are the critical pore diameters for cavitation and equilibrium gas invasion, respectively. The probability that a node connected to the pore provides connection to the vapor phase is denoted with *X*, which is given by

10 $$X=P_{n,e}+\left(P_{g,e}-P_{n,e}\right)\cdot \left(f_{0}+\left(1-f_{0}\right)\cdot \left(1-{\left(1-X\right)}^{z-1}\right)\right)$$

The probability *P*_tr,e_(*p*, *z*, *f*_0_) that a randomly selected pore can empty via gas invasion is related to *X* expressed as

11 $$P_{\mathrm{tr},e}=1-\left(1-f_{0}\right){\left(1-X\right)}^{2\left(z-1\right)}$$

The distribution function for empty pores ψ_e_(*x*, *p*) is given by

12 $$\psi_{e}\left(x,p\right)=\{\begin{array}{ll} 0, & x<x_{g}\\ P_{\mathrm{tr},e}\left(p,z,f_{0}\right)\cdot \phi \left(x\right), & x_{g}\leq x<x_{c}\\ \phi \left(x\right), & x_{c}\leq x \end{array}$$

Finally, eq 2 along with eqs 7 and 12 is used to compute the adsorption and desorption transitions. The adsorbed film volume *V*_af_(*x*, *P*) and all critical pore diameters can be determined using the kernels for N_2_ sorption at 77 K (see for details, ref 47). More specifically, the kernels are produced by combining the thermodynamic models of Bonnet and Wolf on thermally activated condensation–evaporation in cylindrical pores^41,59^ with the cylindrical Lennard-Jones 10-4-3 potential for surface–fluid interaction developed by Siderius and Gelb.^60^ It is proven in ref 47 that the kernels produced in this way are in excellent agreement with those obtained using NLDFT.

## Results and Discussion

### Inverse Problem Solution

To extract the structural descriptors, including the PSD, ϕ­(*x*), the average pore connectivity, ⟨*z*⟩, and the fraction of surface pores, *f*_0_, we solved an inverse problem. For this, it is more convenient to modify eq 2 to

13 $$V_{a}\left(p\right)=\int_{0}^{\infty }K\left(p,x,z,f_{0}\right)\phi \left(x\right)dx$$

with the use of generalized kernels for adsorption

14 $$K_{a}\left(p,x,z,f_{0}\right)=V_{p}\left(x\right)P_{tr,f}\left(p,z,f_{0}\right)+V_{af}\left(x,P\right)\left(1-P_{tr,f}\left(p,z,f_{0}\right)\right)$$

and desorption

15 $$K_{d}\left(p,x,z,f_{0}\right)=V_{p}\left(x\right)\left(1-P_{\mathrm{tr},e}\left(p,z,f_{0}\right)\right)+V_{\mathrm{af}}\left(x,P\right)P_{\mathrm{tr},e}\left(p,z,f_{0}\right)$$

Since we sought to extract a discrete PSD, we first defined a diameter space, [*x*_min_, *x*_max_], of resolution d*x* = (*x*_max_ – *x*_min_)/(*N* – 1), with *N* being the number of discrete pore sizes. Accordingly, we replaced the integral in eq 13 by the sum

16 $$V_{a}^{\left(\exp\right)}\left(p_{i}\right)=V_{\left(a,i\right)}^{\left(\exp\right)}=\sum_{j=1}^{N}K_{\mathrm{ij}}\phi_{j}$$

with

17 $$V_{\left(a,i\right)}^{\left(\exp\right)}=\{\begin{array}{ll} V_{\left(a,i\right)}^{\left(\exp,\mathrm{ads}\right)} & 1\leq i\leq N_{a}\\ V_{\left(a,i\right)}^{\left(\exp,\mathrm{des}\right)} & N_{a}+1\leq i\leq N \end{array}$$

and

18 $$K_{\left(\mathrm{ij}\right)}^{\left(\exp\right)}=\{\begin{array}{ll} K_{\left(\mathrm{ij}\right)}^{\left(\mathrm{ads}\right)} & 1\leq i\leq N_{a}\\ K_{\left(\mathrm{ij}\right)}^{\left(\mathrm{des}\right)} & N_{a}+1\leq i\leq N \end{array}$$

where *N*_a_ are pressure points for adsorption and *N*_d_ = *N* – *N*_a_ are pressure points for desorption.

The kernels *K*_(*ij*)_^(ads)^ and *K*_(*ij*)_^(des)^ were determined using complex expressions that depend on the relative pressure, *p*_*i*_/*p*_0_, as well as the main structural properties of the pore network: the unknown PSD ϕ at the discrete points *x*_*j*_ (*j* = 1, ···, *N*), the pore network connectivity *z*, and the hierarchy factor *f*_0_. Accordingly, we employed an iterating scheme to determine *f*_0_, *z*, and ϕ, minimizing the following general nonlinear least-squares problem

19 $$r_{\theta }\left(\phi \right)=\frac{1}{2}{{∥V}^{\left(\exp\right)}-K\left(f,z,x,\phi \left(x\right)\right)\phi ∥}^{2}$$

The methodology presented in these two sections was applied to two porous materials—random Vycor porous glass, with a pore volume of 0.23 cm^3^/g, and KBS-A with a higher pore volume of 0.4 cm^3^/g. The parameters obtained by the inverse problem solution, which are presented in Table 1, revealed that these two materials have similar PSDs and pore connectivities but substantially different fractions of boundary pores *f*_0_. To assess whether this set, in which all topological features are represented by only ⟨*z*⟩ and *f*_0_, is sufficient for diffusion modeling, we combined theoretical predictions with experiments using PFG NMR.

**Table 1:** Results of Inverse Problem Analysis for Vycor Porous Glass and KBS-A along with Experimentally Measured and Theoretically Predicted Pore-Space Tortuosities

sample	⟨*x*⟩^a^	*f* ^b^	⟨*z*⟩^c^	τ_exp_ ^d^	τ_ST_ ^e^	τ_EMA,SH_ ^f^
Vycor	8.4	1.0 × 10^–4^	3.30	5.3	5.3	5.3; 5.3/5.3
KBS-A	7.0	6.0 × 10^–2^	2.95	3.0	4.7	2.9; 3.1/2.6

a Average pore diameter, nm.

b Effective hierarchy factor *f*, resulting from solution of the inverse problem.

c Average pore connectivity.

d Tortuosity factor from PFG NMR experiments.

e Tortuosity factor predicted by following the Stinchcombe model.^51^

f Tortuosity factor predicted by EMA;^61^ Hashin–Shtrikman lower/upper bounds.^55^

### Transport Prediction via Theoretical Modeling

It is well known that the effective pore diffusivity, *D*_pore_, of a fluid through the pore network is related to the respective effective conductivity σ_e_ of current through its electrical analogue.^24,25^ Thus, *D*_pore_ can be determined by various rigorous or effective medium approximation (EMA) methods, given a network of cylindrical pores of defined pore size distribution ϕ­(*x*) and network connectivity *z*.^52–54^ For the special case of an infinite Bethe network, Stinchcombe has proposed a significant improvement of EMA using a complex integral equation based on series expansions with an error of (*z* – 1)^−5^.^51^

Consistent with the structural model applied in the sorption analysis, we employed SDBL and used Stinchcombe’s theory to calculate the network conductivity.^51^ Although this model is defined for integer connectivities, we allowed for fractional values of *z*, analogous to the approach used for gas sorption. This is justified, at least in part, by earlier studies showing that, in disordered networks, the connectivity distribution has a minor effect on effective conductivity, while the average coordination number is the primary determinant.^62^ By interpreting conductivities in terms of diffusive resistances, we obtained the effective diffusivity and tortuosities, as shown in Table 1 (for more details, see the Supporting Information). Notably, the theory predicted nearly identical values for Vycor and KBS-A, which is consistent with the observed similarities in PSDs and pore connectivity between the two materials. In the next section, we show that this prediction, however, is inconsistent with the experimentally measured diffusivities.

### Pulsed-Field Gradient NMR

Molecular self-diffusion of *n*-hexane in the bulk state and in mesoporous glasses was probed using the PFG NMR technique under equilibrium conditions, i.e., in the absence of any gradient in the chemical potential across the samples.^63^ The method was based on direct measurements of the molecular mean-squared displacements (MSD) along the direction of the applied magnetic field gradients over specified diffusion times *t*_d_.^64,65^ In particular, for normal diffusion, the measured intensity *S* of the spin-echo signal is related to self-diffusivity *D* as

20 $$S\left(q,t_{d}\right)=S\left(0,t_{d}\right)e^{-q^{2}Dt_{d}}$$

where *q* is the wave vector controlled in the experiment.

The resulting diffusion attenuations *S*(*q*, *t*_d_) exhibited a biexponential form, with two diffusivities corresponding to diffusion in excess bulk *n*-hexane surrounding the porous material and in the pore space. The monolithic form of the porous glasses precluded any appreciable molecular exchange between *n*-hexane molecules in the inter- and intraparticle spaces, justifying the use of a biexponential model. The diffusivities obtained from the fits were *D*_0_ = 3.9 × 10^–9^ m^2^/s for bulk hexane and *D*_pore_ = 0.69 × 10^–9^ m^2^/s and *D*_pore_ = 1.22 × 10^–9^ m^2^/s for *n*-hexane in Vycor porous glass and KBS-A, respectively. Despite their structural similarity, PFG NMR diffusion experiments revealed pronounced differences in *n*-hexane diffusivities, and hence tortuosities *D*_0_/*D*_pore_, as reported in Table 1. Notably, the tortuosity measured for Vycor porous glass is in perfect agreement with the literature reports.^66^

### Beyond Statistical Disorder

To explain the observed difference in the diffusion behavior between the two materials, the notable disparity in the fractions of surface pores, *f*_0_, warrants a closer examination. We hypothesize that the unusually high fraction in KBS-A, revealed by the inverse problem solution, reflects the specific organization of the pore space. This may arise if the distribution of pore sizes shows long-range spatial correlations rather than behaving like a completely random (statistically disordered) system. For instance, if a cluster containing a chain of large pores is connected to the external gas phase, as illustrated in Figure 4A, gas invasion into this chain at already relatively high pressures may expose a large number of smaller, otherwise internal, pores to the gas phase. In this context, the fraction of surface pores obtained from the inverse problem should be interpreted as an effective parameter, *f* = *f*_0_ + *f*_sc_, where *f*_sc_ represents the fraction of pores connected to large-pore chains that are themselves connected to the external gas phase. Since the emptying of such clusters is pressure-dependent, *f*_sc_ is an effective quantity reflecting this process.

**Figure 4:**
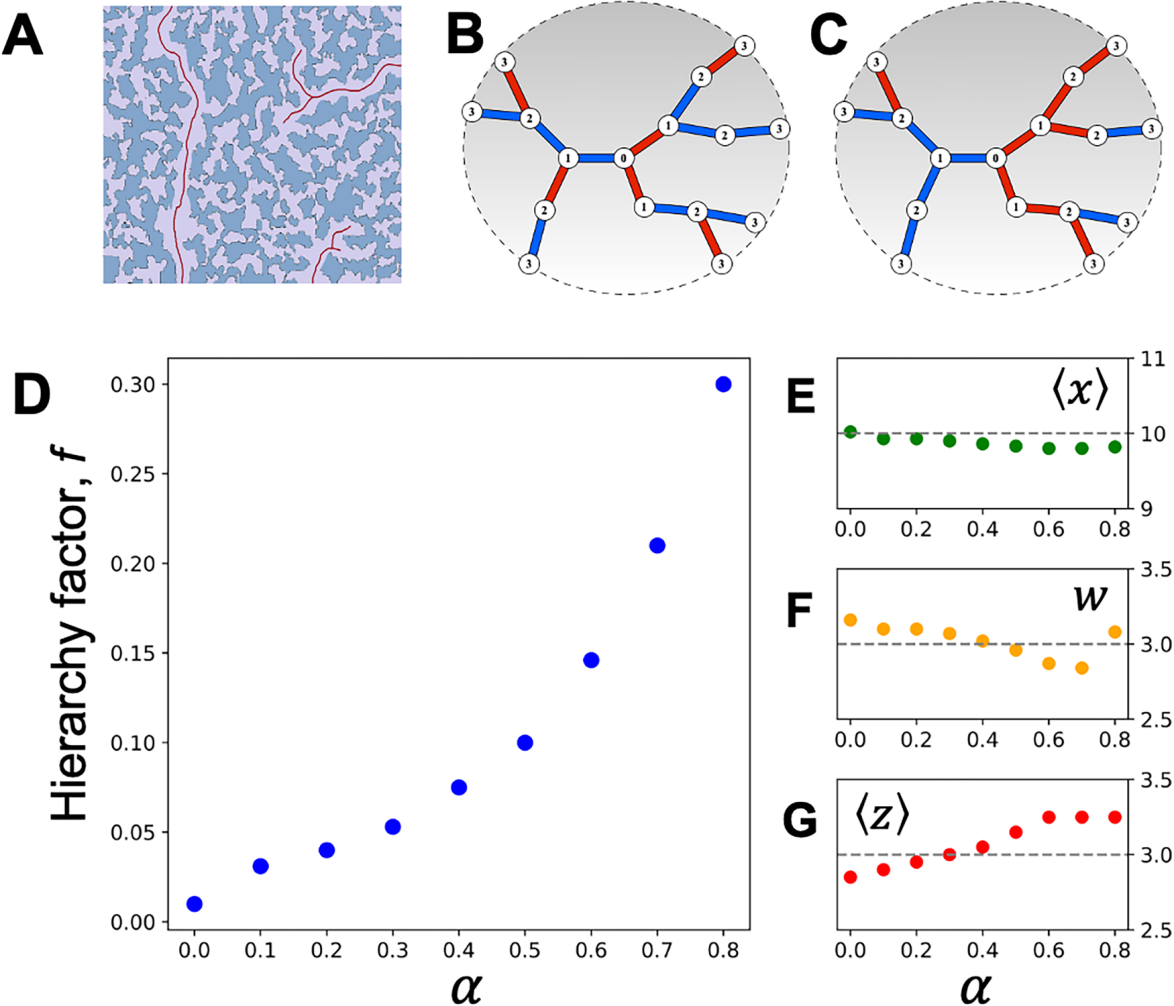
Impact of structural correlations on sorption-derived structural descriptors. (A) Two-dimensional schematic representation of a correlated random pore network. Clusters of large, interconnected pores are highlighted by solid lines. During desorption at relatively high pressures, these clusters expose a substantial fraction of otherwise internal pores to the gas phase, illustrating how structural correlations can enhance the effective surface connectivity of the pore network. (B, C) Two topologically identical SDBL structures with correlation factors α = 0 and α = 0.7, respectively. Bonds colored red and blue indicate pores filled with gas and liquid phases, respectively. Structural correlations strongly influence the phase configurations, leading to the formation of larger single-phase clusters in the correlated pore networks. (D) Effective surface pore or hierarchy factor *f* extracted from sorption isotherms of correlated structures shown as a function of correlation parameter α. (E–G) Other structural descriptors, including the pore size distribution ϕ­(*x*), represented by the mean pore size ⟨*x*⟩ and distribution width *w*, and the average connectivity ⟨*z*⟩, remain nearly constant with increasing α, emphasizing that structural correlations primarily affect *f*.

To explore how structural correlations influence the inverse problem solution, we employed a correlated SDGW model. In contrast to statistical disorder, pore diameters *x* assigned to each bond were correlated with that *x*_par_ of their parent pores formed during generation process as

21 $$x=\alpha x_{\mathrm{par}}+\epsilon$$

where α is the correlation factor assuming values from 0 to 1 and ϵ is the random Gaussian variable with the average ⟨*x*_cor_⟩ = ⟨*x*⟩(1 – α) and dispersion σ_cor_ = σ­(1 – α^2^). This construction scheme resulted in correlated structures with preserved normal PSD, i.e, with the same average pore size ⟨*x*⟩ and dispersion σ, thereby allowing direct illustration of the correlation effects.

We generated various structures with different correlation factors α to compute gas sorption isotherms for pore networks with *z* = 3. We found that these correlations directly influence phase coexistence by producing larger clusters of pores filled with the same phase, as illustrated in Figure 4B,C. Figure 4D–G shows the results of inverse problem analysis. Notably, Figure 4D demonstrates that the extracted effective fraction of surface pores *f* varies strongly with α, while other parameters remain nearly constant. Thus, structural correlations primarily affect *f*, with a clear tendency for increasing *f* with increasing structural correlations.

### TEM-Based Structural Analysis

To verify experimentally the occurrence of specific structural motifs, which could give rise to the observed difference in *f* for two materials, we performed TEM studies. Representative microscopy and their binary images (Supporting Information) for Vycor porous glass and KBS-A are shown in Figure 5A,C, respectively. The binary images were utilized for the computations of the chord length distributions (CLDs) and further for obtaining the PSDs.^67^

**Figure 5:**
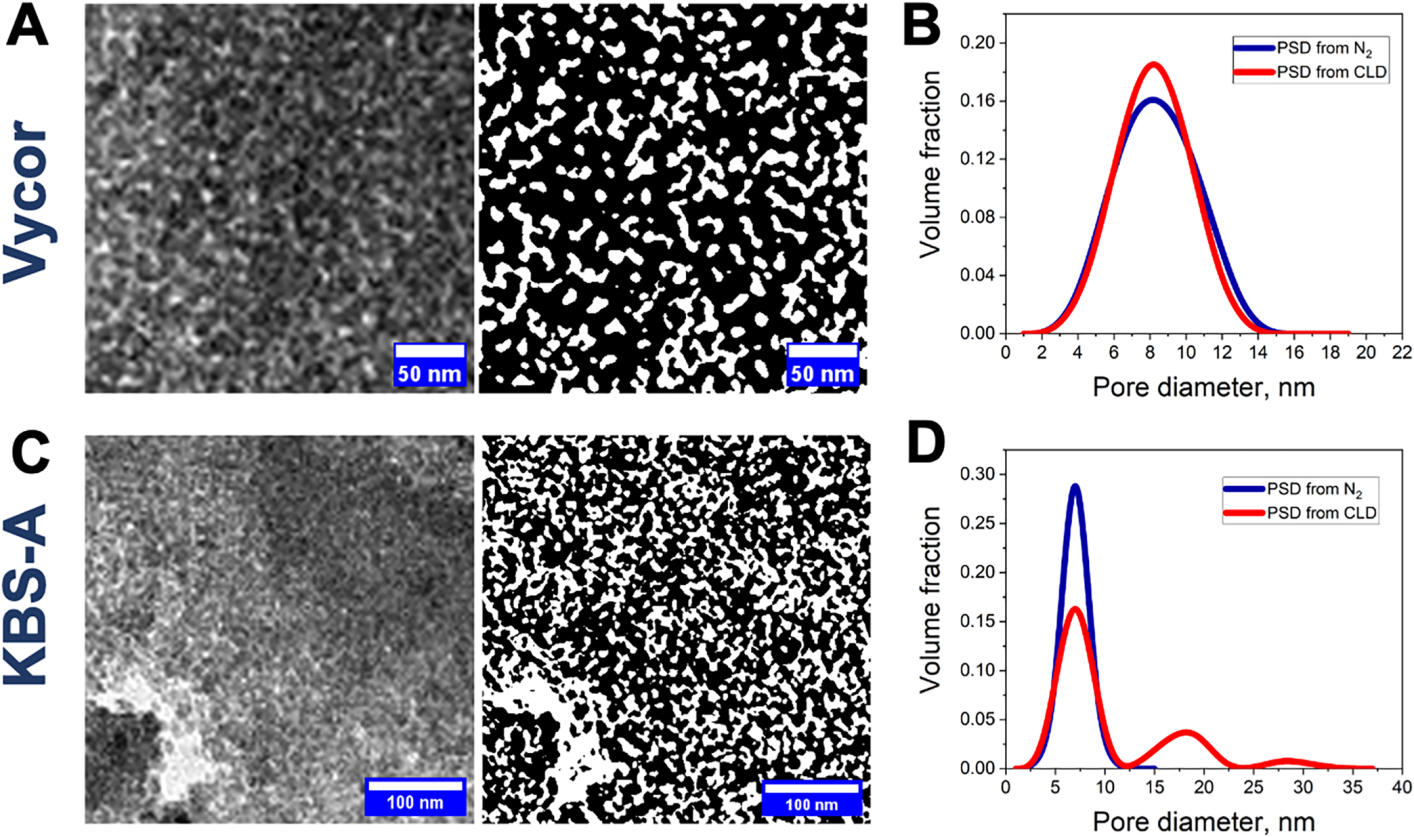
TEM-based pore structure characterization of mesoporous glasses. (A, C) TEM images (left) and the corresponding binary images (right) of Vycor porous glass and KBS-A, respectively. (B, D) Pore size distributions obtained from chord length distribution analysis (red) compared with those derived from nitrogen adsorption (blue) for Vycor porous glass and KBS-A, respectively.

The chord length distribution (CLD) describes the probability of finding chords of a specific length when a geometric object is randomly intersected with lines. For the case of a binary image obtained from image analysis of an electron micrograph, one can measure the CLD on either the pore or solid phase of the image. For the case of pore chord length measurements, it has been previously shown that the PSD is related to the CLD, *A*(*l*) by the following integral equation

22 $$A\left(l\right)=\frac{\int_{0}^{\infty }x^{-1}A_{0}\left(x\right)\phi \left(x\right)dx}{\int_{0}^{\infty }x^{-1}\phi \left(x\right)dx}$$

where **A**_0_ is a kernel producing the CLD in long cylinders of size *x*. We sought to extract a discrete PSD by first defining a diameter space, [*x*_min_, *x*_max_], of resolution d*x* = (*x*_max_ – *x*_min_)/(*N* – 1), with *N* being the number of discrete cylinder sizes. Accordingly, we replaced the integral by the sum

23 $$A^{\exp}\left(l_{i}\right)=\frac{\sum_{j=1}^{N}A_{0j}\phi \left(x_{j}\right)x_{j}^{-1}}{\sum_{j=1}^{N}\phi \left(x_{j}\right)x_{j}^{-1}}$$

Subsequently, the following nonlinear least-squares problem must be minimized

24 $$r_{A}\left(\phi \right)=\frac{1}{2}{∥A^{\left(\exp\right)}-\frac{A_{0}\left(x\right)\phi \left(x\right)x^{-1}}{\phi \left(x\right)x^{-1}}∥}^{2}$$

The latter equation was solved by employing the same methodology used to solve eq 19 above.

Notably, the PSDs resulting from N_2_ sorption and TEM analysis for Vycor porous glass shown in Figure 5B,D were found to be almost identical, confirming the validity of the algorithms used to analyze the TEM data. In contrast, the PSD of KBS-A derived from CLD revealed a bimodal distribution: one mode (referred to as the *primary* porosity) aligned with the PSD obtained from gas sorption, while the other (referred to as the *secondary* porosity) corresponded to significantly larger pore sizes. The secondary pores were also identified using mercury intrusion (see Figure S1 in the Supporting Information). The cumulative number PSD obtained from CLD analysis (see Figure S2 in the Supporting Information) revealed the number fraction of secondary pores *f* ≈ 0.05 in good agreement with the *f*-value from gas sorption. Thus, the high *f*-value observed in gas sorption indicates the presence of a hierarchical pore structure.

It is intriguing that the large pores revealed by TEM and mercury intrusion are not visible in gas sorption. There might be, in our opinion, two reasons for this observation. Large cylindrical pores above ca. 30 nm are difficult to detect with nitrogen sorption at 77 K because capillary condensation occurs close to the bulk saturation pressure. For the smaller pores around 20 nm, specific pore geometry may play an essential role. Noting strong corrugation of these pores seen in the TEM images, it is reasonable to assume that condensation and evaporation transitions are more accurately described by the kernels for spherical pores. The NLDFT kernels for silica surfaces obtained by Ravikovitch and Neimark^68^ reveal that the upper closure point of the hysteresis loop in Figure 3B corresponds to the critical pore diameter of about 20 nm. Hence, the large pores seen by the TEM analysis cannot be resolved by using nitrogen sorption. Most spectacularly, however, the occurrence of the additional network of larger pores is reflected in the elevated value of *f*, which is notably larger than genuine *f*_0_. In this regard, it appears that *f* is indicative of the hierarchical structure organization, hence it may be referred to as the hierarchy *f*-factor to be distinguished from other definitions of the hierarchy factors.^13,69^

### Transport in Structurally Correlated Networks

Having revealed a hierarchical, two-level pore structure in KBS-A, transport modeling can now be adapted using two-phase transport theories. In the first step, the diffusivity within the primary porosity, *D*_pore,h_, and, where applicable, within the second porosity, *D*_pore,s_, was evaluated as described above.

We next considered the case of diffusion in inhomogeneous materials (such as hierarchical porous materials). Due to the direct analogy between molecular diffusion and conduction, we can treat the case of diffusion in inhomogeneous porous structures as that of conduction in two-phase materials. In this case, several expressions based on EMA methods and upper/lower bounds of effective conductivity on composite materials can be used to extract the respective value of *D*_pore_ for the entire inhomogeneous pore structures.^55–57,70^

In particular, the most popular Hashin–Shtrikman lower and upper bounds for three-dimensional isotropic materials are given by

25 $$D_{\mathrm{pore},L}=\left\langle D\right\rangle -\frac{\varphi_{1}\varphi_{1}{\left(D_{2}-D_{1}\right)}^{2}}{\left\langle \mathrm{D̅}\right\rangle +2D_{1}}$$

and

26 $$D_{\mathrm{pore},L}=\left\langle D\right\rangle -\frac{\varphi_{1}\varphi_{1}{\left(D_{2}-D_{1}\right)}^{2}}{\left\langle \mathrm{D̅}\right\rangle +2D_{2}}$$

with

27 $$\left\langle D\right\rangle =\varphi_{1}D_{1}+\varphi_{2}D_{2}$$

and

28 $$\left\langle \mathrm{D̅}\right\rangle =\varphi_{2}D_{1}+\varphi_{1}D_{2}$$

Here, φ_*i*_ and *D*_*i*_ are the relative volume fractions and effective diffusivities of phase *i* in the composite structure (φ_1_ + φ_2_ = 1), respectively. In what follows, we considered that phase 1 is the homogeneous mesoporous matrix (primary porosity), whose effective diffusivity was obtained by the methods described earlier, and phase 2 is the dispersed phase (secondary porosity) that contains large pores where pore diffusivity is practically equal to its bulk value, i.e., *D*_2_ = *D*_0_. With the terms introduced earlier, it is evident that φ_2_ = *f*_*v*_ and φ_1_ = 1 – *f_v_*, where *f*_*v*_ is the volume fraction of the secondary porosity accessible from TEM or mercury intrusion measurements. Finally, we substituted *D*_1_ = *D*_pore,h_, since this variable corresponds to the pore diffusivity of the homogeneous phase in the inhomogeneous porous material.

An improvement to the Hashin–Shtrikman bounds has been proposed by Bruggeman and Landauer and corresponds to the effective medium approximation (EMA) for two-phase, three-dimensional isotropic materials.^25,57^ Following the present terminology, this equation is written as follows

29 $$\left(1-f_{v}\right)\frac{D_{\mathrm{pore},h}-D_{\mathrm{pore}}}{D_{\mathrm{pore},h}+2D_{\mathrm{pore}}}=f_{v}\frac{D_{0}-D_{\mathrm{pore}}}{D_{0}+2D_{\mathrm{pore}}}$$

by assuming *D*_pore,h_ and the bulk diffusivity *D*_0_ for the primary and secondary porosities, respectively. The tortuosity τ can finally be found as τ = *D*_0_/*D*_pore_. As shown in Table 1, this approach yields excellent agreement between predicted and experimentally measured tortuosities and highlights the importance of structural correlations in determining diffusive transport.

## Conclusions

Our results demonstrate that, in structurally disordered porous solids, phase transitions are governed primarily by the average connectivity of the pore space, with the detailed topology playing only a minor role. When combined with sufficiently broad pore size distributions, the same principle also extends to diffusive transport. This insight allows us to establish disordered Bethe lattices as a unifying model that captures the essential structural properties controlling both sorption thermodynamics of nonequilibrium states within the hysteresis region and diffusive transport.

Importantly, we show that the framework is not limited to statistically disordered systems but also applies to materials with spatially correlated pore architectures including hierarchical structures. Overlooking such correlations has likely hindered the clear identification of structure–transport relationships. By incorporating structural descriptors directly accessible from gas sorption, namely the improved pore size distributions ϕ­(*x*), the average connectivity ⟨*z*⟩, and the hierarchy parameter *f* capturing structure deviation from purely random, we established a robust platform for predictive transport modeling.

Beyond its methodological advances, this framework opens new avenues for systematically exploring the relationship between the structure and functionality in nanoporous materials, extending well beyond the transport phenomena. The structural descriptors accessible through gas sorption, which provide a fundamentally richer representation of pore architecture, enable robust correlations with a broad range of properties, including elasticity, heat transfer, and more. Ultimately, this approach establishes a powerful foundation for the rational design of porous solids with tailored performance with far-reaching implications for catalysis, separation, energy storage, and pharmaceutical processing.

## Supplementary Material



## Notes

Additional details on binarization of TEM images, diffusion modeling in homogeneous porous media, cumulative pore size distribution from TEM analysis, and mercury intrusion data (PDF)
