# Supplementary material for: Predicting Structure and Transport in Disordered Mesoporous Materials via Cooperative Phase Transitions
Source: ACS Mater Au. 2025 Dec 25;6(2):425–36. doi: 10.1021/acsmaterialsau.5c00213 (PMC12983104; doi:10.1021/acsmaterialsau.5c00213)
Supplement: Supplementary file 1 [file mg5c00213_si_001.pdf]

Supporting Information

Predicting Structure and Transport in Disordered Mesoporous Materials via Cooperative Phase Transitions

Georgiy Baroncha

Felix Bloch Institute for Solid State Physics, Leipzig University, Linn\'estr. 5, 04103 Leipzig, Germany

Eustathios S. Kikkinides

Department of Chemical Engineering, Aristotle University of Thessaloniki, 54124 Thessaloniki, Greece

Theresa Paul

David Poppitz

Dirk Enke

Institute for Chemical Technology, Leipzig University, Linn\'estr. 3, 04103 Leipzig, Germany

Rustem Valiullin

Felix Bloch Institute for Solid State Physics, Leipzig University, Linn\'estr. 5, 04103 Leipzig, Germany

valiullin@uni-leipzig.de

1. Binarization of TEM images

From TEM studies it is evident that obtained TEM micrographs are grey-level images and must be properly transformed into binary ones using appropriate threshold methodologies. Global thresholding techniques cannot deal efficiently with images that have an unevenly illuminated background as is the present case. In this case, local thresholding is suggested, where the threshold is computed for each pixel according to the image characteristics within a window of radius r (in pixel units) around it.

Hence, in the present work, local thresholding procedures have been employed to transform grey-level to binary (black and white) images. More specifically, the imageJ software has been employed applying several local threshold methods, including, Bernsen's and mid-gray method, producing practically the same results on input TEM images [1]. To validate the local-thresholding methodology, we employed it first on Vycor TEM mages, for which we have detailed and well-documented microscopic characterization information from earlier studies [2-4].

The produced binary images have a porosity around 0.28 in accord with previous studies on this material as well as with N_2 physisorption data at 77 K (0.32) presented in previous sections. Furthermore, higher order statistical properties such as two-point auto-correlation function, chord and mass chord length distribution functions are in very good agreement with previously published studies. Thus, we conclude that the local thresholding methodology can be used on other materials with similar synthesis characteristics. Accordingly, we employed the same methodology on KBS-A material. The resulting porosity was around 0.4. which is in good agreement with the respective porosity obtained by N_2 physisorption at 77 K (0.46). In both cases, the porosity from TEM image-analysis is a bit lower than the one obtained from N2 physisorption (relative error ~13%) and this is attributed mainly to the much smaller samples used in the former compared to the latter experimental technique.

2. Diffusion in homogeneous porous materials

We first focus on diffusion in a homogeneous material using random pore networks. Consider a random network of cylindrical pores with uniform length l. Then, the molar flowrate, F, in a pore, of diameter x is:

$$F = -\frac{1}{4}\pi x^2 D \frac{\Delta c}{l} \tag{2.1}$$

Where D is the diffusivity or diffusion coefficient of the fluid molecules, and Δc is the concentration difference along the pore. For the case of molecular diffusion, it follows that $D=D_0$, which is the bulk molecular diffusivity of the fluid.

Accordingly, in analogy with an electrical network, we define a diffusional pore conductance, $\sigma(x)$, as:

$$\sigma(x) = x^2 \cdot D_0 \tag{2.2}$$

Then, it has been well established that the effective diffusivity, D_{pore}, of a fluid through the pore network is directly related to the respective effective network conductivity, σ_e, of current through its electrical analogue [5, 6].

Thus, D_{pore} can be determined by various rigorous/direct or approximate methods given a network of cylindrical pores of defined number size distribution, $f(x)$, and pore network connectivity, z. Direct methods require the application of Kirchoff's laws at each site of the network and the numerical solution of the resulting set of algebraic equations to obtain the concentration (voltage) profiles in the network. Approximate methods rely on the use of Medium Approximation (EMA) theories, where the effective diffusional conductance, σ_e, of a random resistor network with a conductance distribution, $g(\sigma)$, and connectivity, z, is determined by the solution of the following integral equation [5-7]:

$$\int g(\sigma)d\sigma \left[\frac{\sigma-\sigma_e}{\sigma+\left(\frac{1}{X_p}-1\right)\cdot\sigma_e}\right] = 0 \tag{2.3}$$

Where, $X_p = 2/z$ for regular and $X_p = \frac{1}{z-1}$ for Bethe networks. Furthermore, since it is straightforward to show that for simple pores of constant length, l, conductance distribution is related to number pore size distribution $g(\sigma)d\sigma = f(x)dx$ [7], equation (6.2) becomes:

$$\int f(x)dx \left[\frac{x^2\cdot D_0-\sigma_e}{x^2\cdot D_0+\left(\frac{1}{X_p}-1\right)\cdot\sigma_e}\right] = 0 \tag{2.4}$$

Hence, eq. (8.4) can be employed to determine σ_e given a $f(x)$ and z. Then, applying in addition to EMA the Smooth Field Approximation (SFA) we get the following expression for D_{pore} in a 3D isotropic pore network [5-7]:

$$D_{pore} = \frac{1}{3}\frac{\sigma_e}{\langle x^2 \rangle} \tag{2.5}$$

where $\langle x^2 \rangle$ is the arithmetic average pore size in the network. For the case of liquid diffusion, care must be taken to account for the adsorbed layer that has a small, but non-negligible, contribution to molecular diffusion. In this case, assuming a monolayer of thickness, t_m, the expression for pore diffusional conductance in eq. (.2) becomes:

$$\sigma(x) = \left(1 - \frac{2t_m}{x}\right) x^2 D \tag{2.6}$$

Note that this expression is relatively good valid for large pores, such as considered in this work, but may become incorrect as soon as smaller pores are considered. For the special case of an infinite Bethe network, as in the present wo2rk, Stinchcombe has proposed a significant improvement of EMA using a much more complex integral equation for the effective conductivity, σ_e, of the pore network based on series expansions with an error of the order of $(z\text{-}1)^{-5}$ [8].

More specifically, the effective conductivity, σ_e, of the Bethe network is determined by the following expression [8]:

$$\sigma_e = -\frac{(z-1)}{(z-2))} C'(0) \tag{2.7}$$

where $C'(0)$ is determined by the following integral equation:

$$0 = \int d\sigma g(\sigma)[\tau(\sigma) - C'(0)] + (z-1)I_{210}a_2^0[1 + (z-1)^2 I_{310}a_2^0 + (z-1)I_{220}] + (z-1)I_{310}a_3^0 + I_{410}\left[{}_{z-1}C_2(a_2^0)^2 + (z-1)a_4^0\right] + {}_{z-1}C_2 I_{510}a_2^0 a_3^0 + {}_{z-1}C_3 I_{610}(a_2^0)^3 + O((z-1)^{-5}) \tag{2.8}$$

and:

$$\tau(\sigma) = \frac{\sigma(z-1)C'(0)}{\sigma-(z-1)C'(0)} \tag{2.9a}$$

$$I_{mn0} = \frac{m!}{(n-1)!}\int d\sigma g(\sigma)\frac{\sigma^2[\tau(\sigma)-C'(0)]^{n-1}}{[\sigma-(z-1)C'(0)]^{m+1}} \quad (2.9b)$$

$$a_{n0} = \int d\sigma g(\sigma)\frac{[\tau(\sigma)-C'(0)]^{n}}{n!} \quad (2.9c)$$

As in the EMA equation (8.3), conductance distribution is related to the number pore size distribution by $g(\sigma)d\sigma = f(x)dx$, and σ(x) can be substituted by either eq. (2.2) or (8.6) as before. Furthermore, ${}_xC_y$are the standard binomial coefficients. Eq. (2.7) with the aid of eqs. (2.8) and (2.9), has been employed to determine the effective conductivity and from (2.5) the effective diffusivity D_{pore}, of the homogeneous porous material in the form of a pore network.

3. Mercury Intrusion

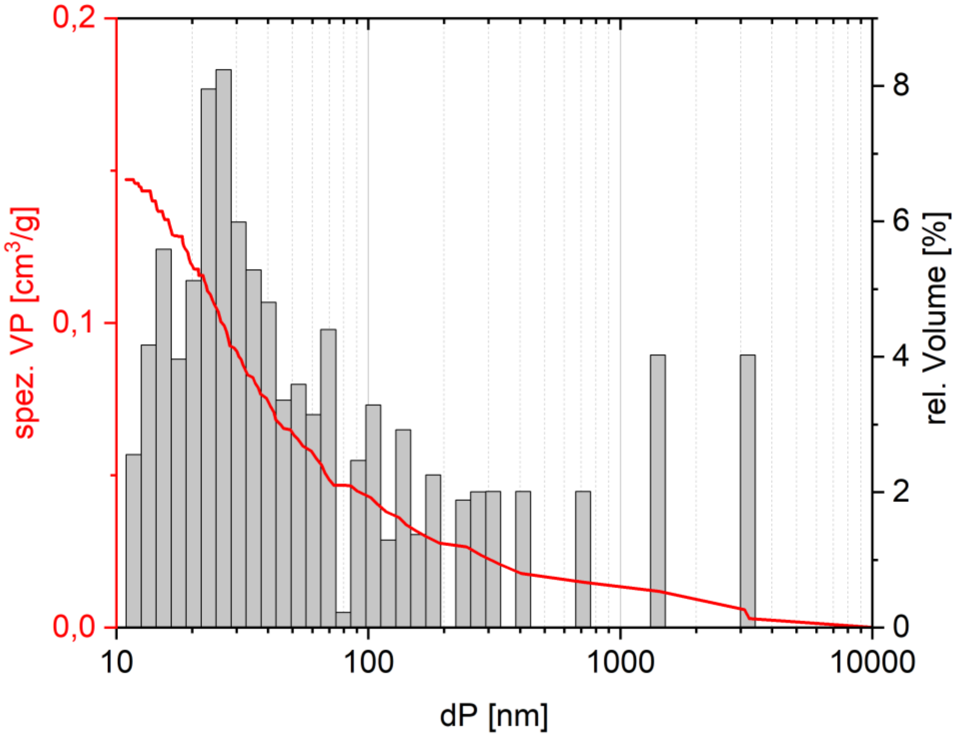

Figure S2. Mercury intrusion data on KBS-A material: The bars show pore size distribution (PSD) and the line is the cumulative PSD.

4. **Chord length analysis**

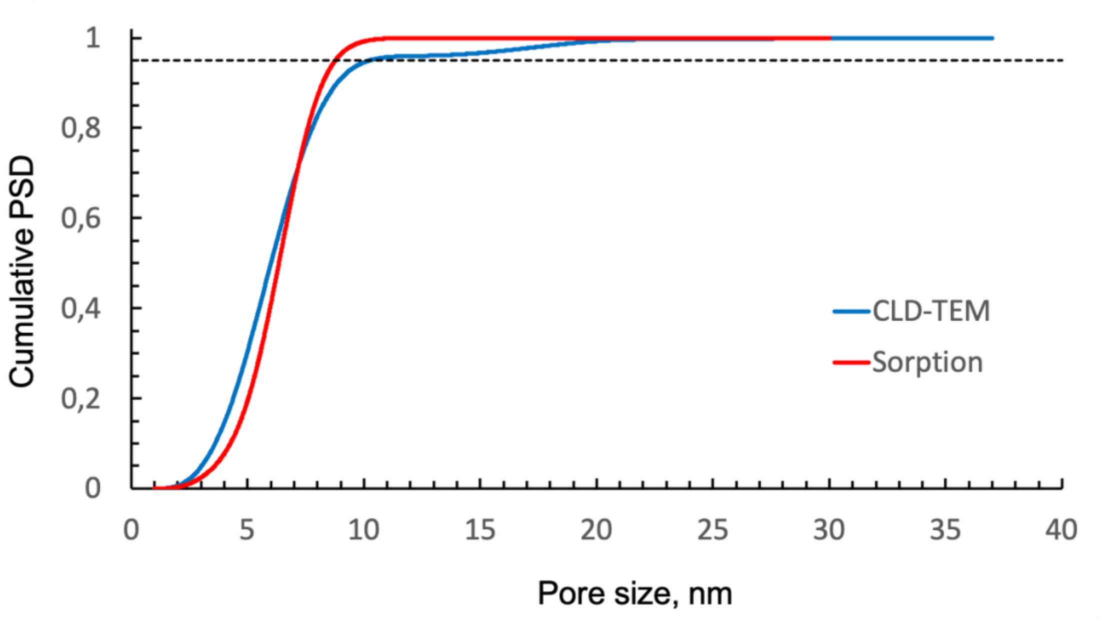

Figure S1. Cumulative number PSD for KBS-A as obtained from the analysis of CLD.

References

1. https://imagej.net/ij/
2. Levitz, P.; Ehret, G.; Sinha, S. K.; Drake, J. M., Porous Vycor Glass - the Microstructure as Probed by Electron-Microscopy, Direct Energy-Transfer, Small-Angle Scattering, and Molecular Adsorption. J Chem Phys 1991, 95, 6151-6161.
3. Levitz, P. Tchoubar, D. Disordered porous solids : from chord distributions to small angle scattering. Journal de Physique I, 2 (6), 771-790 (1992).
4. Gille W., Enke D., and Janowski F., Pore Size Distribution and Chord Length Distribution of Porous Vycor Glass (PVG), Journal of Porous Materials, 9, 221–230, 2002.
5. Brandon C Bukowski, Frerich J Keil, Peter I Ravikovitch, German Sastre, Randall Q Snurr, Marc-Olivier Coppens, Connecting theory and simulation

with experiment for the study of diffusion in nanoporous solids, Adsorption, 27(5) 683-760 (2021).

6. Alexander Schlaich, Jean-Louis Barrat, and Benoit Coasne, Theory and Modeling of Transport for Simple Fluids in Nanoporous Materials: From Microscopic to Coarse-Grained Descriptions, Chem. Rev., 125, 2561−2624 (2025).
7. Burganos V.N., and Sotirchos S.V., Diffusion in Pore Networks: Effective Medium Theory and Smooth Field Approximation, AIChE J., 33(1), 1678-1689 (1987).
8. Stinchcombe R.B., Conductivity and spin-wave stiffness in disordered systems-an exactly soluble model, Journal of Physics C: Solid State Physics, 7(1), 179-203 (1974).
